# 3-(Propan-2-yl­oxy)-1,2-benzothia­zole 1,1-dioxide

**DOI:** 10.1107/S1600536812002413

**Published:** 2012-01-25

**Authors:** Muneeb Hayat Khan, Islam Ullah Khan, Shumaila Younas Mughal, Mehmet Akkurt

**Affiliations:** a Materials Chemistry Laboratory, Department of Chemistry, GC University, Lahore 54000, Pakistan; b Department of Physics, Faculty of Sciences, Erciyes University, 38039 Kayseri, Turkey

## Abstract

In the title compound, C_10_H_11_NO_3_S, the benzisothia­zole ring system is almost planar [maximum deviation = 0.030 (1) Å for the S atom]. The isoprop­oxy group is almost in the plane of the benzisothia­zole ring system [N—C—O—C = 4.5 (2)°] with one of its methyl groups in an anti­periplanar orientation relative to the benzisothia­zole ring system [C—C—O—C = −162.0 (2)°].

## Related literature

For related structures, see: Siddiqui *et al.* (2007 ▶, 2008 ▶); Bassin *et al.* (2011 ▶); Arshad *et al.* (2009*a* ▶,*b* ▶).
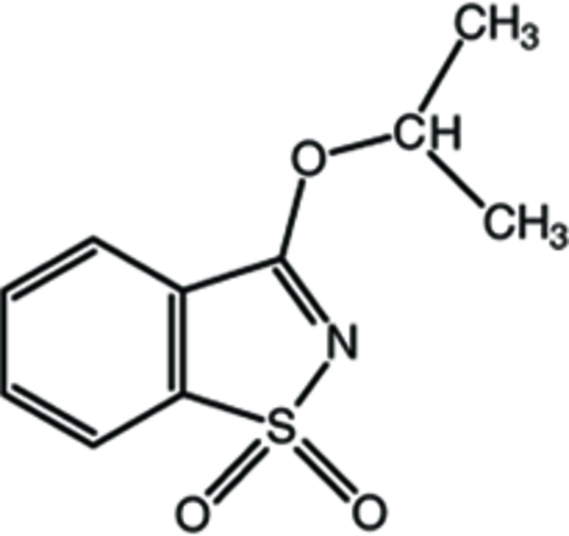


## Experimental

### 

#### Crystal data

C_10_H_11_NO_3_S
*M* *_r_* = 225.27
Triclinic, 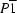

*a* = 8.1899 (3) Å
*b* = 8.8361 (4) Å
*c* = 8.9045 (4) Å
α = 101.624 (2)°
β = 106.694 (1)°
γ = 114.898 (1)°
*V* = 519.89 (4) Å^3^
*Z* = 2
Mo *K*α radiation
μ = 0.30 mm^−1^
*T* = 296 K
0.13 × 0.10 × 0.08 mm

#### Data collection

Bruker APEXII CCD diffractometer
9516 measured reflections
2560 independent reflections
2090 reflections with *I* > 2σ(*I*)
*R*_int_ = 0.020

#### Refinement

*R*[*F* ^2^ > 2σ(*F* ^2^)] = 0.036
*wR*(*F* ^2^) = 0.102
*S* = 1.05
2560 reflections
138 parameters
H-atom parameters constrained
Δρ_max_ = 0.29 e Å^−3^
Δρ_min_ = −0.34 e Å^−3^

### 

Data collection: *APEX2* (Bruker, 2007 ▶); cell refinement: *SAINT* (Bruker, 2007 ▶); data reduction: *SAINT*; program(s) used to solve structure: *SHELXS97* (Sheldrick, 2008 ▶); program(s) used to refine structure: *SHELXL97* (Sheldrick, 2008 ▶); molecular graphics: *ORTEP-3 for Windows* (Farrugia, 1997 ▶); software used to prepare material for publication: *WinGX* (Farrugia, 1999 ▶), *PARST* (Nardelli, 1983 ▶) and *PLATON* (Spek, 2009 ▶).

## Supplementary Material

Crystal structure: contains datablock(s) global, I. DOI: 10.1107/S1600536812002413/ld2045sup1.cif

Structure factors: contains datablock(s) I. DOI: 10.1107/S1600536812002413/ld2045Isup2.hkl

Supplementary material file. DOI: 10.1107/S1600536812002413/ld2045Isup3.cml

Additional supplementary materials:  crystallographic information; 3D view; checkCIF report

## Figures and Tables

**Fig. 1.**
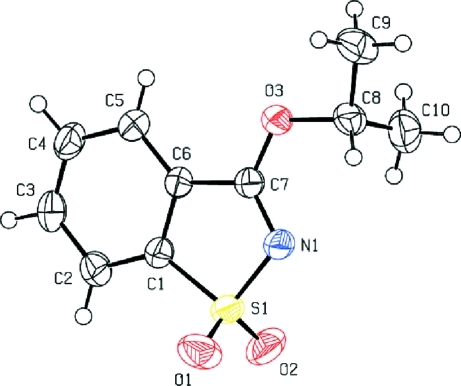
View of the molecule with the atom numbering scheme. Displacement ellipsoids for non-H atoms are drawn at the 50% probability level.

## supplementary crystallographic information

### Comment

The title compound (I) was prepared while synthesizing benzisothiazoles from sodium saccharin. Slight increase in the reaction temperature from 333 K to 353 K give rise to the unexpected product instead of a benzisothiazole derivative.

In the title molecule (Fig. 1), the S atom has a distorted tetrahedral coordination geometry, with S1—O1 = 1.4278 (15), S1—O2 = 1.4264 (16), S1— N1 = 1.6493 (14), S1—C1 = 1.7642 (19) Å, O1—S1—O2 = 117.54 (9), O1—S1—N1 = 109.42 (8), O1—S1—C1 = 110.06 (9), O2—S1—N1 = 109.04 (8), O2—S1—C1 = 112.19 (9) and N1— S1—C1 = 96.54 (8)°. The values of the geometric parameters are in agreement with those observed in related compounds (Siddiqui *et al.*, 2007; Bassin *et al.*, 2011; Arshad *et al.*, 2009*a*,*b*; Siddiqui *et al.*, 2008).

### Experimental

Sodium saccharin (0.5 g m, 2.439 mmol) was placed in a 50 ml round-bottom flask, and 20 ml of the dried DMF were added to it. The mixture was stirred for 5 min. Then iso-propyl iodide (0.243 ml, 2.439 mmol) was added and the mixture was placed under reflux for 3 h at 353 K. After that, the reaction mixture was poured in ice. The precipitate was filtered, washed with ice-cold water, dried and recrystallized from methanol.

### Refinement

All H atoms were positioned geometrically and then treated as riding atoms, with C—H = 0.93 Å (C-aromatic), 0.98 Å (C-methine) and 0.96 Å (*C*-methyl). *U*_iso_(H) =1.2*U*_eq_(C-aromatic, C-methine), and 1.5*U*_eq_(*C*-methyl). The positions of methyl hydrogens were optimized rotationally.

### Crystal data

**Table:**

C_10_H_11_NO_3_S	*Z* = 2
*M**_r_* = 225.27	*F*(000) = 236
Triclinic, *P*1	*D*_x_ = 1.439 Mg m^−^^3^
Hall symbol: -P 1	Mo *K*α radiation, λ = 0.71073 Å
*a* = 8.1899 (3) Å	Cell parameters from 4072 reflections
*b* = 8.8361 (4) Å	θ = 2.6–28.0°
*c* = 8.9045 (4) Å	µ = 0.30 mm^−^^1^
α = 101.624 (2)°	*T* = 296 K
β = 106.694 (1)°	Prism, colourless
γ = 114.898 (1)°	0.13 × 0.10 × 0.08 mm
*V* = 519.89 (4) Å^3^	

### Data collection

**Table:**

Bruker APEXII CCD diffractometer	2090 reflections with *I* > 2σ(*I*)
Radiation source: sealed tube	*R*_int_ = 0.020
graphite	θ_max_ = 28.4°, θ_min_ = 2.7°
φ and ω scans	*h* = −10→10
9516 measured reflections	*k* = −11→11
2560 independent reflections	*l* = −11→11

### Refinement

**Table:**

Refinement on *F*^2^	Primary atom site location: structure-invariant direct methods
Least-squares matrix: full	Secondary atom site location: difference Fourier map
*R*[*F*^2^ > 2σ(*F*^2^)] = 0.036	Hydrogen site location: inferred from neighbouring sites
*wR*(*F*^2^) = 0.102	H-atom parameters constrained
*S* = 1.05	*w* = 1/[σ^2^(*F*_o_^2^) + (0.0509*P*)^2^ + 0.1198*P*] where *P* = (*F*_o_^2^ + 2*F*_c_^2^)/3
2560 reflections	(Δ/σ)_max_ < 0.001
138 parameters	Δρ_max_ = 0.29 e Å^−^^3^
0 restraints	Δρ_min_ = −0.34 e Å^−^^3^

### Special details

**Table:**

Geometry. Bond distances, angles *etc*. have been calculated using the rounded fractional coordinates. All su's are estimated from the variances of the (full) variance-covariance matrix. The cell e.s.d.'s are taken into account in the estimation of distances, angles and torsion angles
Refinement. Refinement on *F*^2^ for ALL reflections except those flagged by the user for potential systematic errors. Weighted *R*-factors *wR* and all goodnesses of fit *S* are based on *F*^2^, conventional *R*-factors *R* are based on *F*, with *F* set to zero for negative *F*^2^. The observed criterion of *F*^2^ > σ(*F*^2^) is used only for calculating -*R*-factor-obs *etc*. and is not relevant to the choice of reflections for refinement. *R*-factors based on *F*^2^ are statistically about twice as large as those based on *F*, and *R*-factors based on ALL data will be even larger.

### Fractional atomic coordinates and isotropic or equivalent isotropic displacement parameters (Å^2^)

**Table:**

	*x*	*y*	*z*	*U*_iso_*/*U*_eq_	
S1	0.51553 (5)	0.65330 (5)	−0.19102 (5)	0.0414 (1)	
O1	0.4815 (2)	0.7249 (2)	−0.31926 (16)	0.0625 (5)	
O2	0.35943 (17)	0.48322 (17)	−0.21499 (18)	0.0616 (4)	
O3	0.85768 (15)	0.97134 (14)	0.24607 (13)	0.0389 (3)	
N1	0.58062 (18)	0.80108 (18)	−0.00718 (16)	0.0386 (4)	
C1	0.7438 (2)	0.6603 (2)	−0.14202 (18)	0.0347 (4)	
C2	0.8040 (2)	0.5704 (2)	−0.2379 (2)	0.0423 (5)	
C3	0.9986 (3)	0.6115 (2)	−0.1638 (2)	0.0472 (6)	
C4	1.1264 (2)	0.7379 (2)	−0.0035 (2)	0.0470 (6)	
C5	1.0640 (2)	0.8267 (2)	0.0923 (2)	0.0402 (5)	
C6	0.8694 (2)	0.78493 (19)	0.02064 (18)	0.0325 (4)	
C7	0.7613 (2)	0.85598 (19)	0.08991 (18)	0.0333 (4)	
C8	0.7475 (2)	1.0339 (2)	0.3210 (2)	0.0418 (5)	
C9	0.9032 (3)	1.2032 (3)	0.4722 (2)	0.0561 (6)	
C10	0.6175 (3)	0.8887 (3)	0.3664 (3)	0.0610 (7)	
H2	0.71820	0.48640	−0.34680	0.0510*	
H3	1.04400	0.55220	−0.22400	0.0570*	
H4	1.25700	0.76420	0.04130	0.0560*	
H5	1.15010	0.91140	0.20090	0.0480*	
H8	0.66590	1.06130	0.23960	0.0500*	
H9A	0.98120	1.17530	0.55260	0.0840*	
H9B	0.84010	1.25380	0.52320	0.0840*	
H9C	0.98750	1.28810	0.43690	0.0840*	
H10A	0.53260	0.77990	0.26810	0.0910*	
H10B	0.53830	0.92470	0.40760	0.0910*	
H10C	0.69860	0.86850	0.45240	0.0910*	

### Atomic displacement parameters (Å^2^)

**Table:**

	*U*^11^	*U*^22^	*U*^33^	*U*^12^	*U*^13^	*U*^23^
S1	0.0302 (2)	0.0471 (2)	0.0359 (2)	0.0212 (2)	0.0067 (2)	0.0023 (2)
O1	0.0688 (9)	0.0846 (10)	0.0389 (7)	0.0538 (8)	0.0105 (6)	0.0162 (7)
O2	0.0311 (6)	0.0500 (7)	0.0705 (9)	0.0097 (5)	0.0136 (6)	−0.0036 (6)
O3	0.0344 (5)	0.0448 (6)	0.0316 (5)	0.0210 (5)	0.0112 (4)	0.0054 (4)
N1	0.0319 (6)	0.0444 (7)	0.0358 (7)	0.0225 (6)	0.0112 (5)	0.0055 (5)
C1	0.0307 (7)	0.0378 (8)	0.0358 (7)	0.0185 (6)	0.0146 (6)	0.0110 (6)
C2	0.0446 (8)	0.0425 (8)	0.0403 (8)	0.0232 (7)	0.0208 (7)	0.0095 (7)
C3	0.0496 (9)	0.0551 (10)	0.0560 (10)	0.0350 (8)	0.0335 (8)	0.0211 (8)
C4	0.0359 (8)	0.0632 (11)	0.0544 (10)	0.0311 (8)	0.0231 (7)	0.0256 (9)
C5	0.0316 (7)	0.0491 (9)	0.0385 (8)	0.0205 (7)	0.0138 (6)	0.0149 (7)
C6	0.0303 (7)	0.0364 (7)	0.0336 (7)	0.0179 (6)	0.0154 (6)	0.0133 (6)
C7	0.0319 (7)	0.0345 (7)	0.0326 (7)	0.0177 (6)	0.0130 (6)	0.0099 (6)
C8	0.0444 (8)	0.0458 (9)	0.0352 (8)	0.0281 (7)	0.0147 (7)	0.0062 (7)
C9	0.0649 (12)	0.0509 (10)	0.0402 (9)	0.0286 (9)	0.0160 (8)	0.0048 (8)
C10	0.0610 (11)	0.0629 (12)	0.0602 (12)	0.0292 (10)	0.0367 (10)	0.0139 (10)

### Geometric parameters (Å, °)

**Table:**

S1—O1	1.4278 (15)	C8—C9	1.508 (3)
S1—O2	1.4264 (16)	C8—C10	1.500 (3)
S1—N1	1.6493 (14)	C2—H2	0.9300
S1—C1	1.7642 (19)	C3—H3	0.9300
O3—C7	1.3101 (18)	C4—H4	0.9300
O3—C8	1.477 (2)	C5—H5	0.9300
N1—C7	1.290 (2)	C8—H8	0.9800
C1—C2	1.379 (2)	C9—H9A	0.9600
C1—C6	1.384 (2)	C9—H9B	0.9600
C2—C3	1.385 (3)	C9—H9C	0.9600
C3—C4	1.377 (2)	C10—H10A	0.9600
C4—C5	1.387 (3)	C10—H10B	0.9600
C5—C6	1.382 (3)	C10—H10C	0.9600
C6—C7	1.478 (3)		
			
O1—S1—O2	117.54 (9)	C1—C2—H2	122.00
O1—S1—N1	109.42 (8)	C3—C2—H2	122.00
O1—S1—C1	110.06 (9)	C2—C3—H3	119.00
O2—S1—N1	109.04 (8)	C4—C3—H3	119.00
O2—S1—C1	112.19 (9)	C3—C4—H4	119.00
N1—S1—C1	96.54 (8)	C5—C4—H4	119.00
C7—O3—C8	117.70 (14)	C4—C5—H5	121.00
S1—N1—C7	109.19 (13)	C6—C5—H5	121.00
S1—C1—C2	130.80 (12)	O3—C8—H8	110.00
S1—C1—C6	106.85 (13)	C9—C8—H8	110.00
C2—C1—C6	122.32 (17)	C10—C8—H8	110.00
C1—C2—C3	116.66 (15)	C8—C9—H9A	109.00
C2—C3—C4	121.7 (2)	C8—C9—H9B	109.00
C3—C4—C5	121.20 (18)	C8—C9—H9C	109.00
C4—C5—C6	117.64 (15)	H9A—C9—H9B	110.00
C1—C6—C5	120.49 (16)	H9A—C9—H9C	109.00
C1—C6—C7	109.38 (15)	H9B—C9—H9C	110.00
C5—C6—C7	130.13 (14)	C8—C10—H10A	109.00
O3—C7—N1	124.94 (16)	C8—C10—H10B	109.00
O3—C7—C6	117.08 (15)	C8—C10—H10C	109.00
N1—C7—C6	117.98 (14)	H10A—C10—H10B	110.00
O3—C8—C9	105.62 (16)	H10A—C10—H10C	109.00
O3—C8—C10	108.91 (16)	H10B—C10—H10C	110.00
C9—C8—C10	113.00 (16)		
			
O1—S1—N1—C7	−114.21 (14)	C2—C1—C6—C7	−179.48 (15)
O2—S1—N1—C7	115.99 (13)	S1—C1—C6—C5	−176.95 (13)
C1—S1—N1—C7	−0.22 (13)	S1—C1—C2—C3	177.44 (14)
O1—S1—C1—C2	−65.92 (19)	C6—C1—C2—C3	−0.4 (3)
O2—S1—C1—C2	66.96 (19)	S1—C1—C6—C7	2.25 (16)
N1—S1—C1—C2	−179.38 (17)	C2—C1—C6—C5	1.3 (3)
O1—S1—C1—C6	112.15 (13)	C1—C2—C3—C4	−1.0 (3)
O2—S1—C1—C6	−114.97 (13)	C2—C3—C4—C5	1.5 (3)
N1—S1—C1—C6	−1.30 (13)	C3—C4—C5—C6	−0.5 (3)
C8—O3—C7—N1	4.5 (2)	C4—C5—C6—C7	−179.86 (16)
C8—O3—C7—C6	−175.82 (13)	C4—C5—C6—C1	−0.8 (2)
C7—O3—C8—C10	76.34 (18)	C5—C6—C7—N1	176.34 (17)
C7—O3—C8—C9	−162.02 (15)	C1—C6—C7—O3	177.57 (14)
S1—N1—C7—C6	1.72 (19)	C1—C6—C7—N1	−2.8 (2)
S1—N1—C7—O3	−178.64 (13)	C5—C6—C7—O3	−3.3 (3)
